# Impact of the KL2/Catalyst Medical Research Investigator Training (CMeRIT) Program on the careers of early-stage clinical and translational investigators

**DOI:** 10.1017/cts.2022.7

**Published:** 2022-01-24

**Authors:** Miriam A. Bredella, Kate M. McGroarty, Lucy Kolessin, Linda F. Bard, Anthony N. Hollenberg, Seward B. Rutkove

**Affiliations:** 1 Harvard Catalyst, The Harvard Clinical and Translational Science Center, Harvard Medical School, Boston, Massachusetts, USA; 2 Department of Radiology, Massachusetts General Hospital, Boston, Massachusetts, USA; 3 Department of Medicine, Weill Cornell Medicine, New York, New York, USA; 4 Department of Neurology, Beth Israel Deaconess Medical Center, Boston, Massachusetts, USA

**Keywords:** KL2, career development awards, research training, translational research, education

## Abstract

The Harvard Catalyst KL2/CMeRIT program is a 2-year mentored institutional career award that includes KL2 grants funded by National Institutes of Health (NIH) and CMeRIT grants funded by Harvard Catalyst nonfederal funds. The purpose of this study was to compare outcomes for early-stage investigators funded by the KL2/CMeRIT program to a group of applicants who were not chosen for support to assess the potential impact of the program on early career outcomes. Career data, including academic promotions, subsequent grant funding, and publication rates, from both successful and unsuccessful 2008–2018 KL2/CMeRIT applicants were compiled throughout the year 2020. Data were obtained directly through outreach to both groups and through assessment of online resources. The cohort comprised 487 individuals, 109 awardees, and 378 nonawardees. Awardees were more likely to be subsequently involved in clinical and translational research than nonawardees (92% vs 75%, *p* < 0.001). A higher proportion of awardees also had achieved academic promotion (81% vs 69%, *p* = 0.016) and subsequent NIH funding (72% vs 58%, *p* = 0.047), while there was no difference in publication rates (*p* = 0.555). Participants in the Harvard Catalyst KL2/CMeRIT program demonstrate greater early career success than nonparticipants though the nonparticipants also fared relatively well.

## Introduction

Harvard Catalyst was founded in 2008 under a grant from the National Institutes of Health (NIH) Clinical and Translational Science Awards (CTSA) Program of the National Center for Advancing Translational Sciences. The CTSA Program is a national consortium of more than 60 research institutions (Hubs) with the overall goal to advance clinical and translation research [1–3]. Harvard Catalyst is a shared enterprise between Harvard University and additional support from partner institutions Harvard Medical School, Harvard T.H. Chan School of Public Health, Beth Israel Deaconess Medical Center, Boston Children’s Hospital, Brigham and Women’s Hospital, Dana-Farber Cancer Institute, and Massachusetts General Hospital. The involvement of partner hospitals is critical to Harvard Catalyst’s ability to achieve its goal to invest in the next generation of clinical and translational researchers in a research landscape focused on team science.

Harvard Catalyst’s KL2 program is a 2-year mentored institutional career award funded by the NIH. In 2012, the CMeRIT program, funded directly by the participating institutions was added to supplement the KL2 grant, given a very high demand for training and funding of early-stage clinical and translational investigators. The content and aim of KL2 and CMeRIT components are identical and thus the two form one joint program. The program provides funding and advanced training in clinical and translational research to senior fellows and early career faculty to pursue a mentored research project.

The expectation is that the research produced in this mentored program will provide the basis for a successful NIH research grant, such as a K08, K23, R01, or other similar grants. In addition to their research project, awardees also pursue educational programs of their choice that provide optimal training focused on their career objectives. This may include Harvard Catalyst-based courses or other suitable translational science education paths. The program also includes dedicated mentoring, grant writing, and other career development opportunities only accessible to KL2/CMeRIT scholars. Ultimately, the program aims to identify talented clinical and translational investigators who would benefit from mentorship and additional training to make the critical leap into independent research.

To determine the impact of the KL2/CMeRIT program on the careers of early-stage investigators, Harvard Catalyst initiated the KL2/CMeRIT Outcomes Project to track and analyze over 10 years’ worth of data from successful and unsuccessful applicants to the program. We hypothesized that individuals who received KL2/CMeRIT awards would demonstrate a higher level of early career success than applicants who were not chosen for funding.

## Materials and Methods

Applications were solicited on the Harvard Catalyst website and at each of the participating hospitals. Each application was reviewed and scored by three reviewers using the NIH scoring system and the applicant was interviewed by the primary reviewer. The top 50% of applications were discussed and scored in a meeting with the entire review committee.

This study includes data from all applicants to the Harvard Catalyst KL2/CMeRIT program from 2008 to 2018, whether they were accepted or not, with a minimum follow-up period of 2 years. In addition to an overall comparison of measures of academic success between program awardees and nonawardees, the study examined the impact of the KL2/CMeRIT awards on women and groups underrepresented in medicine (URiM) as defined by the Association of American Medical Colleges (https://www.aamc.org/initiatives/urm/). The study was exempt from IRB approval.

Sex (male/female/nonbinary) was asked as part of the standard application process. In terms of information on race and ethnicity, at the beginning of the KL2/CMeRIT program, applicants were invited to report such data through a separate survey, outside of the formal application process. Some of the early data were not identifiable and could not be determined to be an applicant or an awardee. In recent years, applicants to the KL2/CMeRIT program were given the option to report this data in the application itself. All applicants were also given the option to not respond to these questions. This project understands that because of the deidentified responses, the project is not able to capture all the URiM data through the program’s history.

### Outcome Measures

The first step of the KL2/CMeRIT Outcomes Project was to define key outcome measures of academic success. These metrics would be used to compare the success of program graduates and applicants who were not accepted into the program. The program chose four keys outcome areas: (1) continued involvement in clinical and translational research, (2) academic promotion, (3) subsequent NIH funding, and (4) publications. The follow-up time was the same for the successful and unsuccessful applicants.

The process of gathering the data for this project began in July 2020. The Harvard Catalyst Evaluation Program, in partnership with the KL2/CMeRIT Program and the Harvard Catalyst Finance Department, identified participant names and populated appropriate outcomes data. Data collectors considered outcomes data reliable if it could be tied to a specific individual with absolute certainty. Data collectors used a combination of unique identifiers such as first, middle, and last names as well as email addresses and employment history sourced through internal Harvard Medical School databases to confirm any external data. External data sources included PubMed, NIH RePORTER, and publicly available information on directories such as LinkedIn and academic biographies from other universities or employers. If there was any uncertainty on the validity of a certain outcome metric for an individual, that individual’s data was not included in the outcome analysis for that particular metric. If an individual applied more than once to the program, only their most recent application record was used for analysis. For a detailed description of applicants who applied multiple time, please see Supplemental Tables.

Exclusion criteria were also set so that only awardees who spent a minimum of 1 year in the program were included in the analysis. One year of program involvement was used as the threshold to ensure the awardees included in the analysis interacted with the program at a level that could potentially yield significant impacts from the program’s offerings, especially the mentoring, grant writing, and other educational components unique to the KL2/CMeRIT program. There were 121 awardees of which 12 (10%) departed the program before 1 year. Of those 11 (92%) did so because of having received additional NIH funding shortly thereafter (generally a K08, K23, or R award). This study focused only on the 109 investigators who completed at least 1 year of the program to help ensure that the specific mentoring and education components provided by the program contributed to their success. Data collection was completed by the end of September 2020.

#### Continued Involvement in Clinical and Translational Research

The category “Continued involvement in clinical and translational research” tracked whether successful and unsuccessful applicants were still involved in clinical and translational research as of July 2020. This project utilized the metric framework developed by the Center for Leading Innovation & Collaboration for the Common Metrics Careers in Clinical and Translational Research metric (*CTSA Common Metric Operational Guideline: Careers in Clinical and Translational Research.* clic-ctsa.org) to determine involvement in clinical and translational research.

KL2/CMeRIT Outcomes Project Data collectors used a combination of publications, funding, and employment data to make a determination of continuous research involvement. If research activity was evident within the year 2020, in any of the roles listed above, that individual was determined to be involved in clinical and translational research.

#### Academic Promotion

Data on academic promotion included if a participant received a promotion after their application to the program. In addition to an individual’s ability to advance in their careers, this project evaluated if awardees and nonawardees were retained within the Harvard academic system. The ability to retain highly talented investigators is essential to the success of the Harvard academic community and will strengthen the ability of the community to engage in critical team science. For those investigators who remained in the Harvard Medical School system, data were sourced from an internal Harvard Human Resources database. For those who had left Harvard, data collectors sourced their promotions data from official online biographies first and if required, from other professional online sources.

#### Subsequent NIH Funding

An important goal of the program is the ability to obtain independent grant funding from the NIH as principal investigator (PI). Therefore, subsequent NIH funding was tracked to indicate if the program made it more likely that a graduate received K, R, or other funding from the NIH as PI. This data were sourced directly from the NIH Research Portfolio Online Reporting Tool (RePORTER). Sourcing data from RePORTER ensured this metric would have a high level of data completion across both groups.

Because it can take several years for an investigator to obtain research funding from the NIH, the project opted to use a smaller pool of awardee and nonawardee data for this metric, limiting the analysis to only those individuals who had applied to the program between the years of 2008–2015. Similarly, only individuals who were reported to be involved in clinical and translational research were included in the subsequent NIH funding metric.

#### Publications

The number of publications provides a measure of research productivity. Data were collected on publication volume of a given investigator as of July 2020. A portion of the data was sourced from Harvard Catalyst Profiles, a searchable online profile of all faculty at Harvard Medical School, Harvard School of Dental Medicine and Harvard T.H. Chan School of Public Health. Harvard Catalyst Profiles links PubMed data to a specific, identifiable researcher, making it easier for data collectors to confirm the overall publication volume for a single investigator. When an individual was not present in Harvard Catalyst Profiles, a data collector made a best effort to find publication data through PubMed or official biographies at other academic institutions. If data collectors were not able to confidently source an investigator’s publications, they were excluded from the analysis. No specific accounting was made for journal impact factor or other measures of “publication success” beyond the simple number of successful publications. Additionally, it was determined that the various career lengths made the overall volume calculations meaningless. Using the first publication date as a starting point, data collectors determined how long a given investigator had been actively publishing, up to July 2020. This data were used to determine an individual’s annual publication rate.

### Statistical Analysis

Chi-square analyses were performed to compare categorical proportions between two or more groups. Two-group comparisons of continuous values were made using the Student’s *t*-tests. For all statistical analyses, *p* < 0.05, two-tailed, was used as an indication of significance.

## Results

The final database included 487 unique individuals of which 109 (22%) were KL2/CMeRIT grant awardees and 378 (78%) were nonawardees.

### Study Cohort

Table 1 summarizes the study cohort by year of award and group status (awardee vs nonawardee). Of note, in 2008, the program did not have formal application process and instead sourced the first cohort from other Harvard Catalyst educational programs.

**Table 1.** Study cohort

Cohort year	Awardees in program > 1 year	Nonawardees	Total
2008	6 (100%)	0	6
2009	8 (11%)	65 (89%)	73
2010	8 (19%)	35 (81%)	43
2011	7 (21%)	26 (79%)	33
2012	12 (32%)	26 (68%)	38
2013	5 (14%)	30 (86%)	35
2014	14 (39%)	22 (61%)	36
2015	10 (20%)	40 (80%)	50
2016	15 (22%)	53 (78%)	68
2017	12 (24%)	39 (76%)	51
2018	12 (22%)	42 (78%)	54
Total	109 (22%)	378 (78%)	487

Data are presented as number of individuals and (%).

The total number of men and women in the cohort was nearly equal with 245 (50%) women and 252 (50%) men. Of the 245 women applicants to the program, 50 (20%) were awarded and 195 (80%) were not awarded. Our records do not include any individuals without gender data, with nonbinary responses or from any investigators who chose to not answer the gender question. Of the 59 URiM applicants to the program, 10 (17%) individuals were awarded a KL2/CMeRIT grant and 49 (83%) did not receive an award (Table 2).

**Table 2.** Demographics of KL2/CMeRIT awardees and nonawardees

	Awardees	Nonawardees	Total
Women	50 (20%)	195 (80%)	245 (50%)
Men	59 (24%)	183 (76%)	242 (50%)
URiM	10 (17%)	49 (83%)	59 (12%)
Non-URiM	99 (23%)	329 (77%)	428 (88%)

Data are presented as number of individuals and (%).

URiM, underrepresented in Medicine.

#### Continued Involvement in Clinical Translational Research

**Fig. 1.**
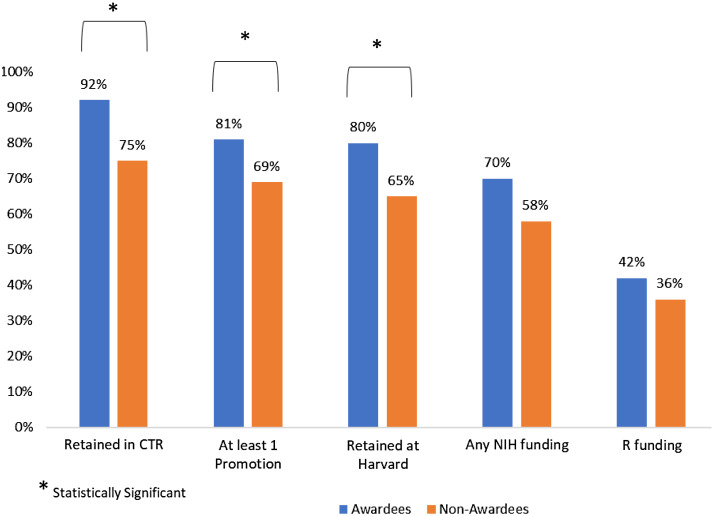
Outcomes of all KL2/CMeRIT awardees versus nonawardees. CTR, clinical and translational research.

Continued involvement in clinical and translation research is a main goal of the program. The relationship between a person’s interaction with the program and their continued involvement in clinical and translational research proved to have the greatest positive relationship. Of the 109 awardees, 100 (92%) have been retained in clinical and translational research whereas of the 363 nonawardees with reliable data, 271 (75%) have been retained in clinical and translational research (*p* < 0.001) (Fig. 1).

#### Academic Promotion

Of the 109 awardees, 88 (81%) had received at least one academic promotion after being admitted to the program. Of the 373 nonawardees with reliable data, 257 (69%) had received at least one promotion after their application to the program (*p* = 0.016). Five nonawardees had unreliable data and were excluded from the analysis.

Of the 109 awardees, 85 (78%) were retained within the Harvard academic system and of the 376 nonawardees with reliable data, 245 (65%) were retained at Harvard (*p* = 0.034) (Fig. 1).

#### Subsequent NIH Funding

As mentioned previously, of the 121 awardees, 11 (9%) received NIH funding within the first year of the program and were not included in the analysis. In comparison, of the 378 nonawardees, 6 (2%) received grant funding within the first year of their unsuccessful application to the KL2/CMeRIT program.

Using the previously described inclusion criteria for subsequent NIH funding (applicants from 2008 to 2015 and remaining involved in clinical and translational research), 65 awardees were included in this analysis. Of those, 47 (72%) received subsequent NIH funding. Of the 180 nonawardees who met inclusion criteria, 105 (58%) received subsequent NIH funding (*p* = 0.047).

Twenty-nine awardees (45%) received subsequent NIH R awards versus 65 (36%) of nonawardees (*p* = 0.230) (Fig. 1).

#### Publications

Data collectors were able to collect publication data on all 109 program awardees, but only 361 nonawardees had viable and available publication data. The mean (±SD) number of publications for awardees was 2.94 ± 2.59 publications per year and the mean number of publications for nonawardees was 2.77 ± 2.9 publications per year (*p* = 0.555).

Because there was no formal application process in 2008 and the first cohort was sourced from other Harvard Catalyst educational programs, there were no unsuccessful applicants in that year. We therefore performed a separate analysis excluding the 2008 awardees. After excluding the 2008 cohort, 95 of the 103 awardees (92%) have been retained in clinical and translational research whereas 271 of 363 nonawardees (75%) with reliable data have been retained in clinical and translational research (*p* < 0.001). Of the 103 awardees, 83 (81%) had received at least one academic promotion after being admitted to the program, and of the 373 nonawardees with reliable data, 257 (69%) had received at least one promotion after their application to the program (*p* = 0.020). Using the previously described inclusion criteria for subsequent NIH funding, 60 awardees were included in this analysis. Of those, 42 (70%) received subsequent NIH funding. Of the 180 nonawardees who met inclusion criteria, 105 (58%) received subsequent NIH funding (*p* = 0.108). The mean (±SD) number of publications for awardees with reliable data was 2.91 ± 2.62 publications per year and the mean number of publications for nonawardees was 2.77 ± 2.9 publications per year (*p* = 0.626).

### Impact of the KL2/CMeRIT Program on Women and Individuals Underrepresented in Medicine

**Fig. 2.**
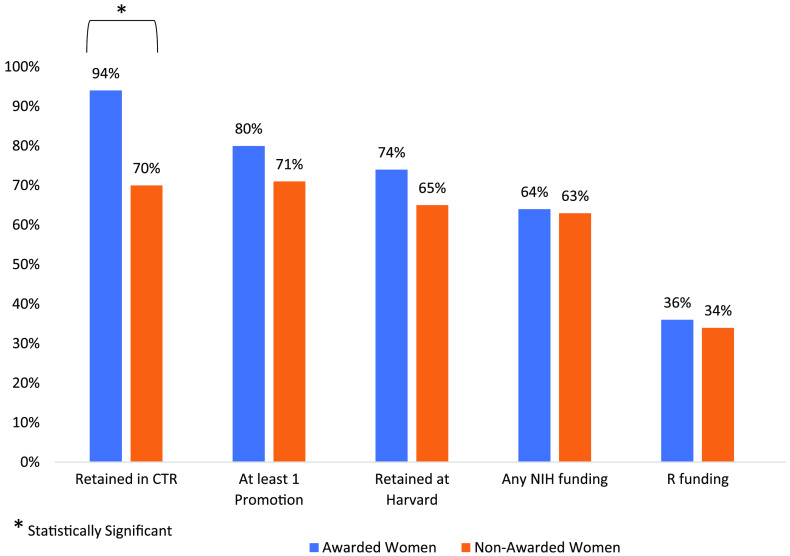
Outcomes of women KL2/CMeRIT awardees versus nonawardees. CTR, clinical and translational research.

Of the 50 awarded women, 47 (94%) were retained in clinical and translational research whereas of the 184 nonawarded women with reliable data, 129 women (70%) were retained in clinical and translational research (*p* < 0.001). However, none of the other outcomes were significantly different between funded and nonfunded women, although funded women fared better overall than unfunded women (Fig. 2).

When comparing the results of the 10 URiM awardees to the 47 nonawarded URiM individuals with reliable data, there were no significant differences between the groups in continues involvement in clinical and translational research (80% vs 77%, *p* = 0.787), academic promotion (60% vs 58%, *p* = 0.897); retention at Harvard Medical School (80% vs 57%, *p* = 0.178), subsequent NIH funding (60% vs 50%, *p* = 0.689), NIH R funding (20% vs 35%, *p* = 0.520), or publication rate (2.03 ± 0.88 vs 2.89 ± 2.96 publications per year, *p* = 0.101). However, this analysis is limited by a considerably smaller sample size than the other populations and the statistics may be misleading due to the small sample size.

## Discussion

In this study, we attempted to discern the impact of obtaining funding through our KL2/CMeRIT program on the future academic successes of early-stage investigators compared to a group of applicants to the program who were not funded. While this is not a controlled experiment, in that we do not know what would have happened had the unselected been funded or vice versa, the results support the general notion that funding through the program helped maintain the individuals in the field of clinical and translational research. Critically, the number of faculty continuing in clinical and translational research was very high (92%) and was also seen in women who are traditionally underrepresented as PIs [4]. Women who were awarded a KL2/CMeRIT grant were more likely to remain involved in clinical and translational research compared to women who were not awarded (94% vs 70%, *p* < 0.001). In addition, when looking at the entire cohort, awardees were more likely to be promoted, retained in the Harvard system, and were more likely to receive subsequent NIH funding.

The overall higher rate of retention in clinical and translational research, higher promotion, and funding rates could be due to a number of factors and may not simply be due to the actual quality or effectiveness of the program. Clearly, having funding provides protected time to pursue research, but also creates a positive incentive to pursue such research, which then leads to subsequent funding. In addition, the KL2/CMeRIT grant provides access to educational offerings, such as individual advisory committees, grant writing courses, biostatistics, and many others, some of which are not accessible to nonawardees.

Obtaining funding sooner means that individuals enrolled in the program had a head-start leading to a simple temporal advantage. Moreover, it is possible that our selection process identified individuals with superior potential, and thus the greater success in this group could reflect those inherent personal characteristics and not the strength of the program. Nevertheless, we were surprised and happy to learn that a large percentage of nonfunded individuals did not give up on their quest to pursue research and were also ultimately successful. This certainly attests to the perseverance of many individuals and of course implies potential additional success had they been funded.

Given that we only had a limited amount of data on each person, we did not attempt to identify additional features that could indicate the impact of this funding on broader academic success. For example, we did not specify the specific types of R or other grants that were received, nor did we attempt to gauge the impact factor of the journals in which publications occurred or the citation rate for those articles, both of which might provide more granular data on the success of the candidates. Similarly, the follow-up time on the candidates was limited. While some of the earlier scholars had follow-up data extending out as much as 10 years, more recent graduates had data extending only out 3 years. Trends over time in the composition of a given class of scholars could influence the number of participants and thus could provide somewhat misleading data. For example, in recent years we have implemented targeted initiatives to encourage URiM individuals to apply to the program and have been able to achieve a very high rate of URiM enrollment. Therefore, our data might be biased to the relatively few URiM awardees who completed the program in its earliest years.

This work has intentionally only focused on the academic outcomes and not personal or social findings. For example, we do not know if those who were not selected delayed beginning a family nor do we have any sense of overall job satisfaction or general level of happiness. Such measures are clearly critical, since academic advancement and success only represents a single, relatively narrow compositional thread in one’s life story. Nevertheless, since the program is an academic one, focusing on future academic success is appropriate. Also, we were unable to compare the KL2/CMeRIT program to another grant program, which might have provided valuable information on the impact of the unique components of the KL2/CMeRIT program on scholars’ future successes. Additionally, for applicants that applied more than once we only included the final application. Another limitation of our study was that we were not blinded to the status of the applicants while completing the analysis. A major strength of our study is the detailed longitudinal assessment of awardees and nonawardees allowing us to assess the impact of the program on a cohort of Harvard early career faculty.

In conclusion, the Harvard Catalyst KL2/CMeRIT program is successful in advancing clinical and translational researchers, especially women. Participants in the program generally fared better than those who did not as measured by a number of criteria. However, the interpretation of those differences remains uncertain and could be due to factors beyond the quality of the program itself. Further study of this and other CTSA training programs from across the country over more extended periods of time will be needed to fully understand the impact of these programs on the long-term success of clinical and translational researchers.

## Supplementary material

For supplementary material accompanying this paper visit https://doi.org/10.1017/cts.2022.7. click here to view supplementary material
