# Supplementary material for: Impact of the KL2/Catalyst Medical Research Investigator Training (CMeRIT) Program on the careers of early-stage clinical and translational investigators
Source: J Clin Transl Sci. 2022 Jan 24;6(1):e16. doi: 10.1017/cts.2022.7 (PMC8889229; doi:10.1017/cts.2022.7)
Supplement: Supplementary file 1 [file S2059866122000073sup001.docx]

**Supplemental Tables**

**Supplemental Table 1**: Number of successful and unsuccessful applicants who applied more than once to the program

| \|  \| **Awarded** \| **Non-Awardees** \| \| --- \| --- \| --- \| \| Second Application \| 22 \| 72 \| \| Third Application \| 3 \| 10 \| \| Fourth Application \| 1 \| 1 \|   **Supplemental Table 2: Number of non-first applications per application year. Data are presented as number of individuals**   \| **Cohort Year** \| **Second Application** \| **Third Application** \| **Fourth Application** \| \| --- \| --- \| --- \| --- \| \| 2010 \| 8 \| 0 \| 0 \| \| 2011 \| 8 \| 1 \| 0 \| \| 2012 \| 5 \| 3 \| 0 \| \| 2013 \| 10 \| 0 \| 0 \| \| 2014 \| 6 \| 0 \| 0 \| \| 2015 \| 7 \| 1 \| 0 \| \| 2016 \| 14 \| 2 \| 0 \| \| 2017 \| 14 \| 3 \| 0 \| \| 2018 \| 13 \| 2 \| 1 \| \| 2019 \| 9 \| 1 \| 1 \| |
| --- | --- | --- | --- | --- | --- | --- | --- | --- | --- | --- | --- | --- | --- | --- | --- | --- | --- | --- | --- | --- | --- | --- | --- | --- | --- | --- | --- | --- | --- | --- | --- | --- | --- | --- | --- | --- | --- | --- | --- | --- | --- | --- | --- | --- | --- | --- | --- | --- | --- | --- | --- | --- | --- | --- | --- | --- |
|  |
